# Effective treatment of apatinib in desmoplastic small round cell tumor: a case report and literature review

**DOI:** 10.1186/s12885-018-4135-x

**Published:** 2018-03-27

**Authors:** Chunyu Shi, Ye Feng, Lei Chao Zhang, Da Yong Ding, Ming Yu Yan, Lu Pan

**Affiliations:** 1 Department of Gastrointestinal colorectal and anal surgery, China-Japan Union Hospital of Jilin University, Changchun, China; 2 Department of Pathology, China-Japan Union Hospital of Jilin University, Changchun, China; 3 Department of Radiology, Changchun Traditional Chinese Medicine Hospital, Changchun, China; 4 Department of Pediatric Immunology, Allergy and Rheumatology, The No.1 Hospital of Jilin University, Changchun, China

**Keywords:** Apatinib, Desmoplastic small round cell tumor, VEGFR-2

## Abstract

**Background:**

Desmoplastic small round cell tumor (DSRCT) is a rare malignant sarcoma with poor prognosis due to lack of effective treatments. Apatinib is a new potent oral small-molecule tyrosine kinase inhibitor, and targets the intracellular domain of vascular endothelial growth factor receptor 2 (VEGFR-2). In this study, we presented a case of intra-abdominal DSRCT which was effectively treated by apatinib.

**Case presentation:**

A 32-year-old man was admitted due to increasing urination frequency and palpable mass in right lower abdomen for 2 months. The mass was resected and diagnosed DSRCT. The patient refused chemotherapy and radiotherapy,and used Chinese medicine only. Six months after the surgery, the patient re-hospitalized due to growing abdominal mass and ascites. Intraperitoneal cisplatin treatment showed little effect. Apatinib was then recommended. Apatinib revealed outstanding effect on reducing mass size and ascites during 2-month treatment. Apatinib therapy continued for additional 2 months, and the patient was in good condition. The only toxicity was hand-food syndrome, which was controllable and well tolerated.

**Conclusion:**

It is the first report that apatinib is effective on DSRCT. This report may provide an additional option for the treatment of metastatic DSRCT.

## Background

Desmoplastic small round cell tumor (DSRCT) is a rare malignant and aggressive tumor. Only 850 such patients were reported in the medical literature [1]. DSRCT was first described by Gerald and Rosai in 1989 [2, 3]. It mainly occurs among children and young adults. No standardized treatment guideline is available nowadays. Current treatment consists of surgical resection combined with chemotherapy, radiotherapy [4]. Although these multimodel therapies, DSRCT still has a poor prognosis, with less than 30% three-year survival rate and only 18% five-year survival rate [5, 6]. Thus, novel therapy is required.

Apatinib (Hengrui Pharmaceutical Co., Ltd., Shanghai, China) is a small molecule tyrosine kinase inhibitor (TKI) and targets vascular endothelial growth factor receptor 2 (VEGFR-2). Apatinib has been proved to be effective and safe in advanced gastric cancer, metastatic breast cancer, esophageal cancer, and non-small-cell lung cancer. Moreover, the drug has shown a substantial potential in multiple solid tumors [7]. However, there is no any report for apatinib in treating DSRCT to day. In this study, we presented a case of intra-abdominal DSRCT, which was effectively treated by apatinib.

## Case presentation

**Fig. 1:**
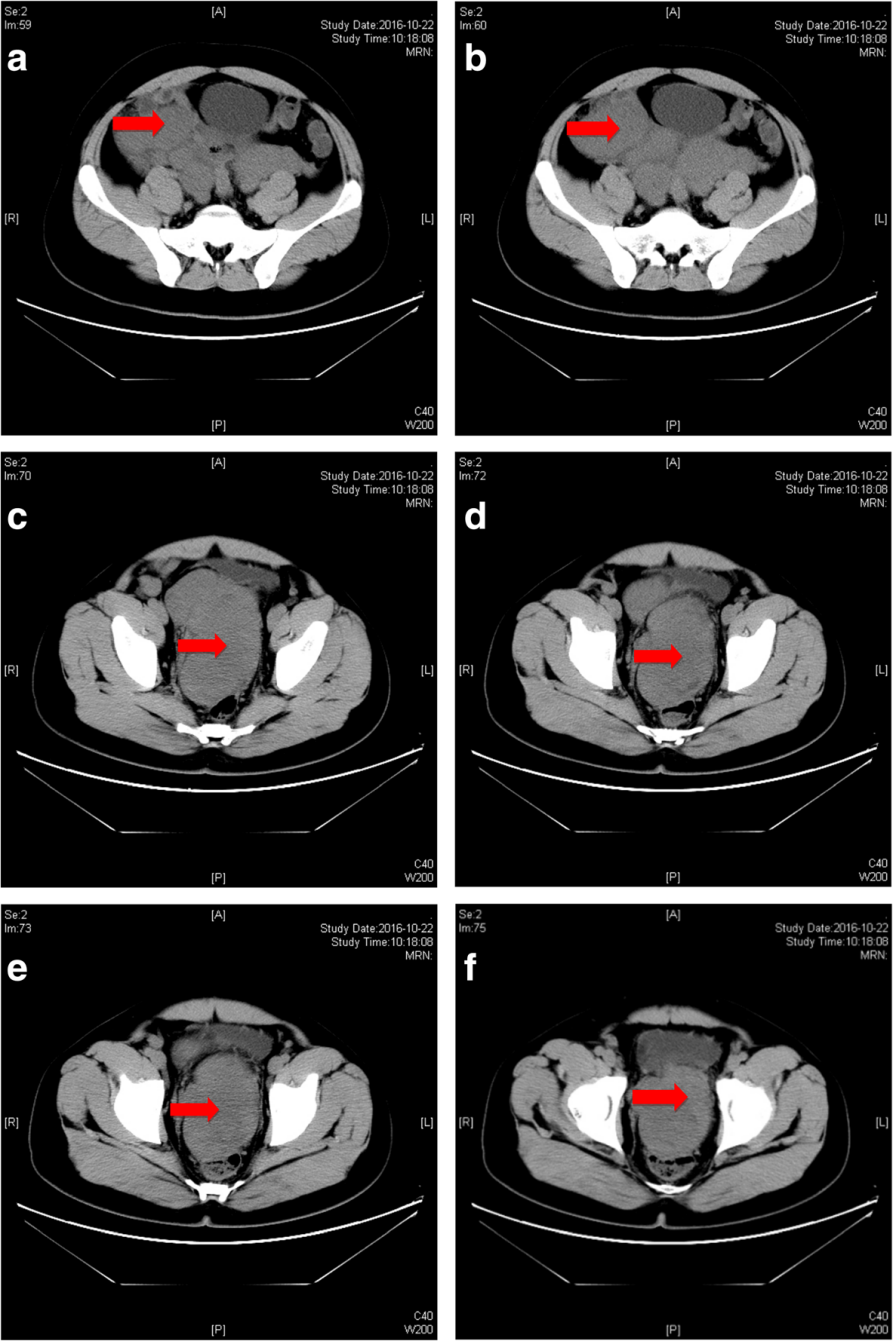
**a**-**f** Abdominal CT revealed a 13.9 × 10.6 × 17.4 cm mass between bladder and rectum

On October 18, 2016, a 32-year-old man was admitted to China-Japan Union Hospital due to increasing urination frequency and palpable mass in right lower abdomen for 2 months. Besides abdominal distension, no other associated symptom was detected. The patient received appendicectomy 12 years ago due to acute appendicitis. Physical examination revealed that the mass had hard texture, unclear boundary, and a low degree of mobility. Abdominal computed tomography (CT) showed a soft tissue density mass measuring 13.9 × 10.6 × 17.4 cm between bladder and rectum, and the mass appeared to originate from mesentery (Fig. 1).

On October 25, the patient was given laparotomy. There were 3 masses between bladder and rectum with firm consistency and gray-white color. The sizes were about 7 × 6 × 5 cm, 8 × 7 × 4 cm, and 6 × 6 × 5 cm. No invasion was found in diaphragm, liver, spleen and pancreas. Dozens of metastatic nodules (0.5 × 0.5 × 1 ~ 5 × 2 × 1 cm) were found on the surface of omentum and mesentery of small and big intestine. All neoplasms were resected and sent for pathological evaluation.

**Fig. 2:**
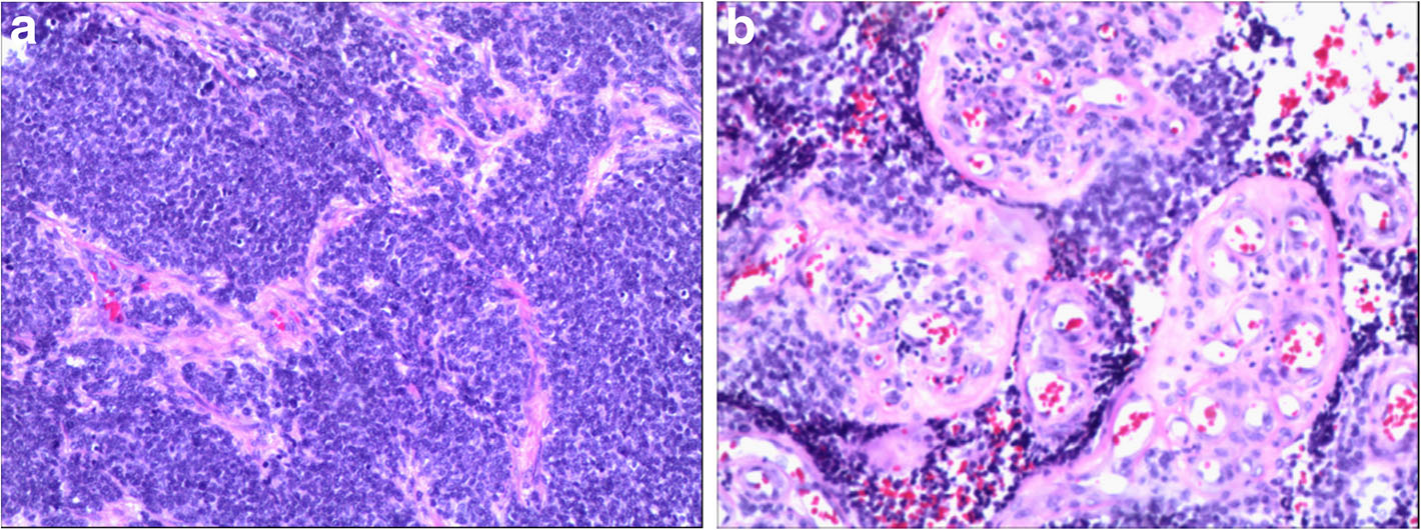
Histological appearance of desmoplastic small round cell tumor (Hematoxylin and Eosin stain, **a**: 100 × magnification, **b**: 200 × magnification)

**Fig. 3:**
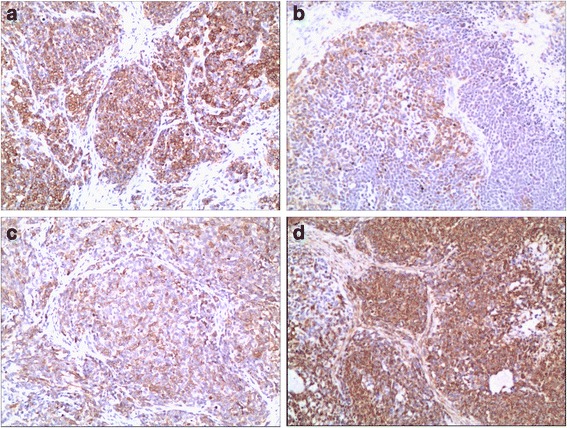
Immunohistochemistry of desmoplastic small round cell tumor (Hematoxylin and Eosin stain, 100 × magnification, **a**: CK, **b**: Desmin, **c**: EMA, **d**: Vimentin)

Microscopic histolology revealed a malignant neoplasm composed of variable sizes of tumor cell clusters distributed in abundant desmoplastic cellular stroma. Tumor cells were undifferentiated and small to medium in size with round/oval hyperchromatic nuclei and inconspicuous nucleoli (Fig. 2). Immunohistochemistry showed tumor cells were positive to CK, epithelial membrane antigen (EMA), desmin, vimentin, CD99, WT1 and neuron-specific enolase (NSE), and negative to actin, CD34, S100, D2–40, and GATA3. Ki-67 proliferation index was 40% (Fig. 3). Cytogenetic analysis demonstrated EWSR1 (22q12) translocation. The pathologic findings were supportive for the diagnosis of DSRCT.

After surgery, the patient had a good recovery, and left hospital on November 4, 2016. The patient received traditional Chinese medicine therapy instead of chemotherapy and radiotherapy because of concerns of the toxicities such as vomiting, nausea, headache, etc.

**Fig. 4:**
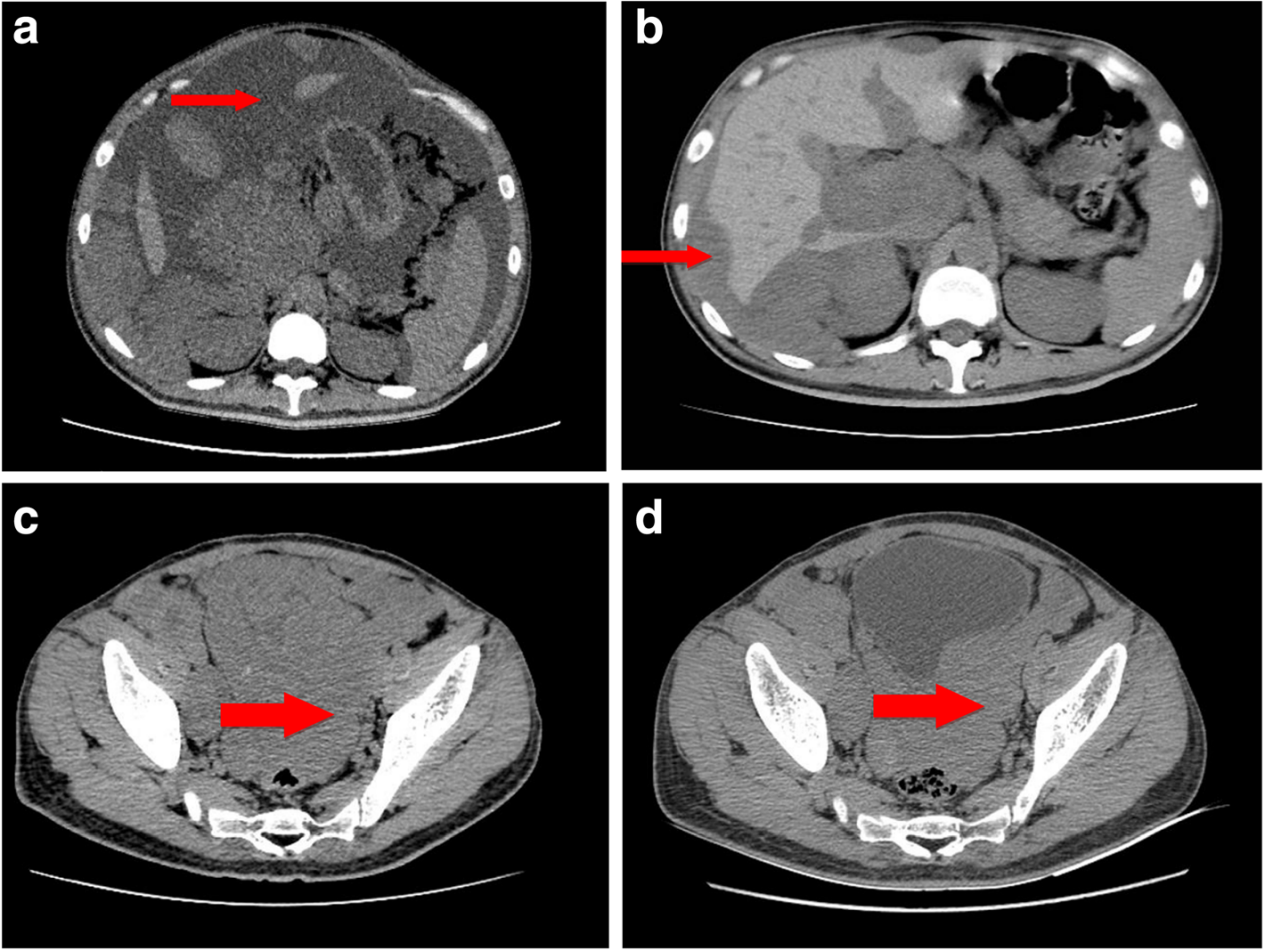
Abdominal CT scans before and after apatinib therapy. **a**. Before apatinib therapy (May 19, 2017), CT scan showed extensive ascites. **b**. After 1 month of apatinib treatment (July 3,2017), ascites decreased significantly. **c**. After 1 month of apatinib treatment (July 3,2017), CT scan showed the mass in front of rectum. **d**. After 2 months of apatinib treatment (August 7, 2017), the mass was smaller than 1 month ago(reduced in size by 40%).The thin and thick arrowheads aim at ascites and metastatic mass in front of rectum, respectively

In May 2017, the patient was hospitalized to our hospital due to abdominal distension, and growing and palpable abdominal mass. Moreover, he suffered breathing difficulty and was hard to lie flat at night.. Serum Carbohydrate antigen(CA125) level was 184.88 U/mL. Abdominal CT scan indicated abdominal metastasis and extensive ascites (Fig. 4a). Ascites was light yellow and clear, and there were malignant cells in it. The patient was given intraperitoneal cisplatin on May 23, 2017. By the end of the treatment, the symptoms had no mitigation and abdominal circumsference and weight was not reduced. Quantitative polymerase chain reaction of resected tissue before was then performed. The results revealed high expression of VEGFR-2. Thus, apatinib-targeted therapy was recommended, and the patient signed informed consent.

On June 4, 2017, apatinib was administered at a dose of 500 mg/d. After one-month therapy, serum CA125 level was reduced to 60.2 U/mL. Symptoms were significantly relieved and body weight and abdominal circumsference were decreased from 66 kg to 50 kg. CT scan showed abdominal ascites diminished significantly (Fig. 4b-c). After one more month of apatinib treatment, serum CA125 level was decreased to normal level (25.3 U/mL). CT scan revealed abdominal mass was further reduced (Fig. 4d). The toxicity the patient experienced was hand-food syndrome. The patient continued to use apatinib as maintenance therapy. Until now, apatinib therapy continued for additional 2 months, and the patient was in good condition.

## Discussion and Conclusions

DSRCT is an extremely rare and highly aggressive malignant neoplasm. Estimated incidence is 0.2~ 0.5 per million people [8]. It typically presents as a large intra-abdominal mass with numerous small peritoneal implants [9]. Extra abdominal DSRCT is rare. However it was reported that some DSRCTs originated from soft tissues and bone [10], testis [11], nasal cavity [12], pleural cavity [13], uterus [14], kidney [15], lung [16] and skull [17]. Liver and lung are the most two common sites of metastases [18]. DSCRT also metastasizes to lymph nodes of the groin, neck, or mediastinum [19].

Clinical manifestations of DSRCT are nonspecific, including abdominal pain, distention, constipation, bowel obstruction, nausea, ascites, and compression symptoms of urinary organs such as urinary disorders. Physical examination may reveal a palpable mass in the abdominal or pelvic cavity. Ultrosonic, CT scan and MRI may reveal multiple tumor nodules.

Microscopically, DSRCT is characterized by nests of small round blue cells separated by desmoplastic stroma which shares features with other round cell tumors such as small cell carcinoma, mesothelioma, Wilms’ tumor, and Ewing’s sarcoma/peripheral neuroectodermal tumors (PNET). The gold standard for the diagnosis of DSRCT is the results of histopathology and cytogenetics. DSRCT simultaneously expresses epithelial (CK and EMA), mesenchymal (vimentin and desmin), and neural (CD56 and NSE) markers [20]. Almost all DSRCTs are positive to WT-1 [21]. Cytogenetic study shows unique (11:22), (p13:q12) translocation which resulted in an active fusion protein involving the Ewing sarcoma (EWS) and Wilms tumor (WT1) genes [4]. The presence of this translocation provides definitive diagnosis for DSRCT [22].

The treatment of DSRCT remains challenging. No standard treatment regimen is available nowadays. Current therapies mainly include surgical therapy, chemotherapy, and radiotherapy [4]. Primarily surgical approach is the optimal scheme. It has been reported that 3-year survival rate in cases with complete tumor resection was 58%, while the number was 0% in nonresectable cases [9]. However, in patients with extraperitoneal metastasis, surgery can not produce any benefit [23]. Owing to peritoneal implantation, microscopic residual disease is often present even after resection. Hyperthermic intraperitoneal chemotherapy (HIPEC) with cisplatin was considered as an effective adjunctive therapy [4]. Currently the most accepted chemotherapy was reported by Kushner et al. [5] in 1996, and the alkylating agents (cyclophosphamide or ifosfamide, along with vincristine and doxorubicin alternating with ifosfamide and etoposide) were similar to those used in Ewing sarcoma. In 2009, Mora et al. published a series of pediatric cases treated with gemcitabine and docetaxel in relapse therapy [24]. The survival benefiting from chemotherapy still outweighs its side effects. Multiple agent chemotherapy is still recommended with aggressive surgery in DSRCT. Radiation therapy in DSRCT is controversial. Goodman observed significant hematological and gastrointestinal toxicities in whole abdominopelvic radiotherpy [25]. While intensity-modulated radiation therapy (IMRT) which was used in the adjuvant setting was beneficial to survival [26]. Immunotherapy of DSRCT is under investigation. About 96% of DSRCT cells express neoantigen B7H3, which is targeted by monoclonal antibody 8H9. A phase I trial of intraperitoneal radioimmunotherapy with ^131^I-8H9 is in process in patients with DSRCT, which showed a good toleration [4].

Magnan et al. reported that VEGFR-2 is overexpressed in DSRCT and that DSRCT xenografts have been shown to be highly responsive to anti-VEGF agents such as bevacizumab [27]. Small molecule TKIs including sunitinib and pazopanib have shown effective in treating DSRCT in some cases [28–30]. These results suggest that small molecule TKIs may block VEGFR and their downstream pathways, which might represent a possible treatment option for advanced DSRCT patients.

In this case, the patient refused to receive chemotherapy at initial treatment and the effect of intraperitoneal chemotherapy with cisplatin at second hospitalization was not obvious. Gene expression analysis showed the patient expressed high level of VEGFR-2. Targeted therapy was then recommended. However, no list of sunitinib and pazopanib in domestic, so we chose apatinib as a third choice.

Apatinib (YN-968D1) is one of the latest small-molecule TKIs targeting VEGFR-2. Some encouraging clinical data have shown that apatinib is effective in treating a variety of solid tumors [7]. However, it was not reported whether apatinib exerts effects in the treatment of DSRCT. Apatinib was successfully applied as a third or fourth line drug in advanced gastic cancer and was approved and launched in China in 2014. It was also currently tested in phase II and III clinical trials in non-small cell lung cancer, breast cancer, hepatocellular carcinoma and colorectal cancer [7]. Hypertension, proteinuria and hand-foot syndrome are the most common adverse effects.

In the present study, the patient developed abdominal metastasis and extensive ascites 8 months after surgery. Apatinib effectively relieved the symptom and reduced ascites and the mass size. The survival time has been over 4 months since the treatment. A longer-term efficacy of apatinib can be expected. Additionally, the patient suffered no severe toxicity except for hand-foot syndrome, which was controllable and well tolerated.

To the best of our knowledge, this is the first report for apatinib in treating DSRCT. High level of VEGFR-2 expression in DSRCT and succefully treatment with sunitinib and pazopanib for DSRCT provide mechanism support for the treatment. Further prospective trials are needed to further confirm the efficacy and safety of apatinib in the clinical treatment of DSRCT.

## Abbreviations

CA125: Carbohydrate antigen 125
CT: Comupted tomography
DSRCT: Desmoplastic small round cell tumor
EMA: Epithelial membrane antigen
HE: Hematoxylin-Eosin stain
HIPEC: Hyperthermic intraperitoneal chemotherapy
IMRT: Intensity-modulated radiation therapy
NSCLC: Non-small-cell lung cancer
NSE: Neuron-specific enolase
PNET: Peripheral neuroectodermal tumours
TKI: tyrosine kinase inhibitor
